# Secondary Hemophagocytic Lymphohistiocytosis in a Post-COVID-19 Patient

**DOI:** 10.7759/cureus.22620

**Published:** 2022-02-26

**Authors:** Sai Samyuktha Bandaru, Ashley Capace, Vishal Busa, Aaron Williams

**Affiliations:** 1 Internal Medicine, Baton Rouge General Medical Center, Baton Rouge, USA

**Keywords:** post-covid-19 manifestations, sars-cov-2, hemophagocytic lymphohistiocytosis (hlh), immune activation, secondary hemophagocytic lymphohistiocytosis

## Abstract

Hemophagocytic lymphohistiocytosis (HLH) is a life-threatening condition caused by excessive immune system activation. HLH can be primary or secondary. Primary HLH is commonly seen in children with underlying genetic mutations, while secondary HLH can be seen at any age. It is usually triggered by inciting factors such as viral infections, patients with underlying rheumatological disease, or malignancies. It has very poor prognosis if left untreated, with survival of only a few months. While there have been around 100 cases of HLH reported during the acute phase of COVID-19 infection, very few post-COVID-19 HLH cases have been reported, only around 35 cases. Here we report a case of a 20-year-old Caucasian male who presented eight weeks after COVID-19 infection with extreme fatigue, fever, lab work concerning for HLH, and a high H score indicating a high probability of HLH. Early identification of HLH following COVID-19 recovery would allow for timely management of the condition.

## Introduction

Hemophagocytic lymphohistiocytosis (HLH) is an extremely aggressive and lethal disorder with varying etiologies. It is categorized as primary and secondary. Primary HLH includes familial/genetic causes, whereas viral infections, underlying malignancies, or rheumatological disorder can trigger secondary HLH [1]. The most common findings seen are fever, pancytopenia/bicytopenia, neurological symptoms such as seizures, change in mentation, ataxia, and lymphadenopathy. Laboratory findings such as marked cytopenia, hyperferritinemia, hypofibrinogenemia, hypertriglyceridemia, and transaminitis are predominantly seen in patients with HLH [2]. Severe acute respiratory syndrome coronavirus-2 (SARS-CoV-2) virus, which causes COVID-19, is a known causative agent of secondary HLH in the acute phase of COVID-19 infection; however, secondary HLH in post-COVID-19 patients is rare [3,4]. Subclinical inflammation with immune dysregulation has been thought to cause secondary HLH in post-COVID-19 patients. Here, we report a case of HLH seen in a patient eight weeks post-COVID-19 infection.

## Case presentation

**Figure 1:**
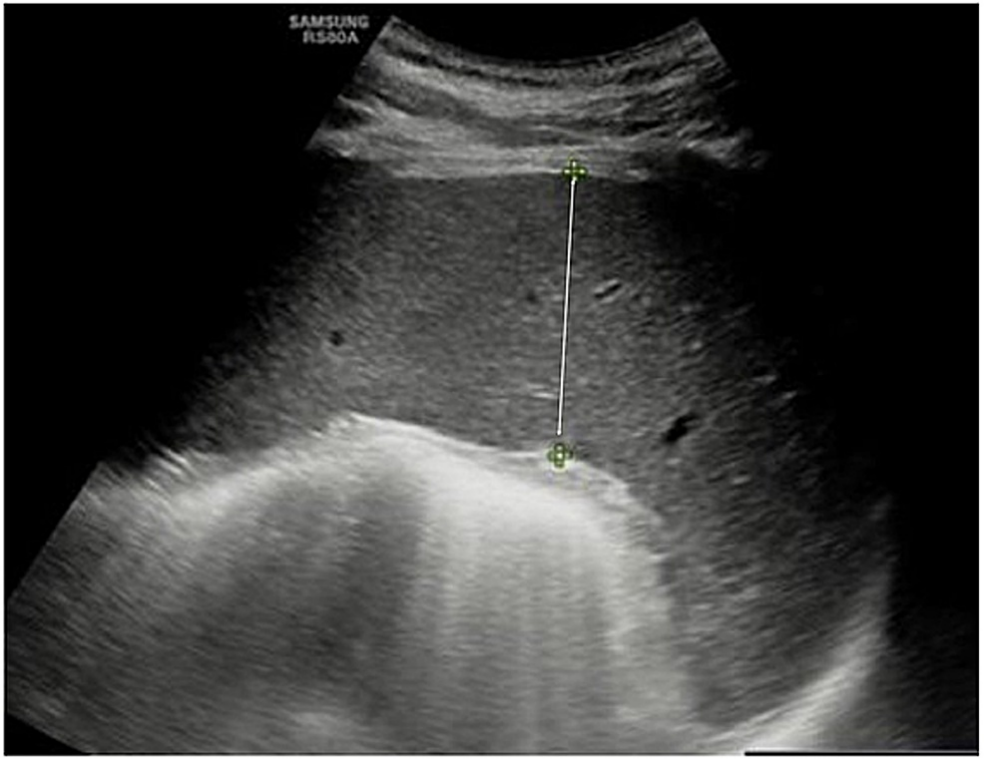
Ultrasound showing splenomegaly.

**Figure 2:**
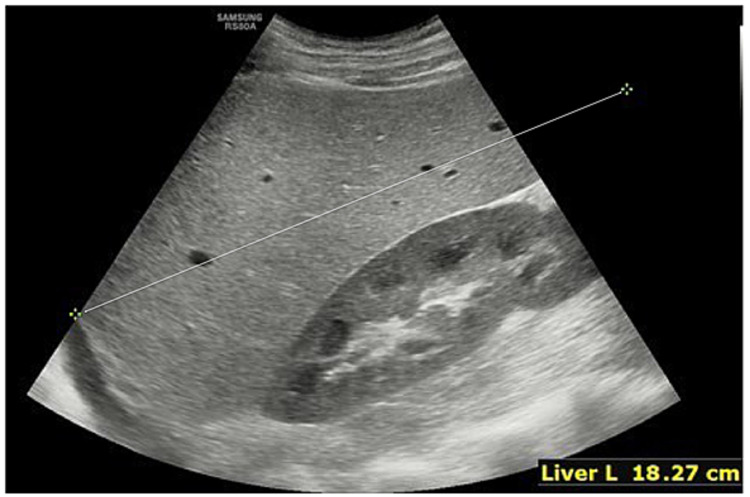
Ultrasound showing hepatomegaly.

A 20-year-old Caucasian male without any significant past medical history presented to our emergency department on October 30, 2021, eight weeks after he had been diagnosed with COVID-19. He had experienced progressively worsening symptoms, including shortness of breath, generalized weakness, and fatigue. His symptoms had started over one week prior to presentation, with fever of 101°F, dizziness, lightheadedness, palpitations, exertional dyspnea, and exercise intolerance. Eight weeks prior to his hospitalization, he had developed fever, cough, nausea, and vomiting. He had presented to an urgent care clinic where he was diagnosed with COVID-19. His COVID-19 infection was uncomplicated and was treated symptomatically without requiring supplemental oxygen or hospitalization. One week prior to the admission, the patient went to urgent care a couple of times for the same symptoms, which were treated as post-COVID-19 respiratory symptoms with steroids and antibiotics without any blood workup obtained. The patient did not have a sore throat, abdominal pain, myalgia, joint pain, skin rashes, or lymphadenopathy. On physical examination, the patient was tachycardic and mildly tachypneic. He was conscious, alert, and oriented. Grossly, the patient appeared pale with conjunctival pallor. He had mild splenomegaly on examination. No skin rashes were noticed. His laboratory workup revealed significant anemia (hemoglobin at 1.8 g/dL), thrombocytopenia (platelet count of 50,000 cells), transaminitis (alanine aminotransferase at 2,058 IU/L and aspartate aminotransferase at 2,000 IU/L), alkaline phosphatase at 86 IU/L, total bilirubin at 1.2 mg/dL, lactate dehydrogenase at 1913 U/L, iron at 216 µg/dL, ferritin at 21,059.8 ng/mL, triglycerides at 69 mg/dL, fibrinogen activity at 344 mg/dL, and a high CD25 level of 1,556. Acute viral hepatitis panels that include hepatitis A, B, and C virus antibodies were non-reactive, and the Monospot screen for Epstein-Barr virus (EBV) was negative. Further imaging with abdominal ultrasonography revealed hepatosplenomegaly with a spleen measuring up to 14.8 cm (Figures 1, 2).

The patient’s H score was 174 points, indicating a 54%-70% probability for HLH [5] (temperature > 101.1°F, +33 points; hepatosplenomegaly, +38; 3 cell lineages, +34; ferritin > 6,000, +50; aspartate aminotransferase > 30, +19). These findings were consistent with and raised concerns for HLH. Hematology/oncology specialists were consulted for further recommendations. Our patient was initiated on etoposide 135 mg/500 mL and high-dose Decadron (20 mg) as per HLH treatment guidelines. The patient’s further response and subsequent laboratory examination results are summarized in Table 1.

**Table 1:** Patient labs before and after treatment.

	On admission	During hospitalization after initiation of treatment		
Laboratory test	Day 1	Day 3	Day 5	Day 7
Hemoglobin, g/dL	1.8	6.4	9.1	9.2
White blood cell count, K/uL	6.12	1.99	1.92	2.12
Platelet count	56,000	41,000	39,000	38,000
Ferritin, ng/mL	21,059.8	5,415.4	2,057.6	1,510.0
Alanine aminotransferase, IU/L	2,058	1094	681	441
Aspartate aminotransferase, IU/L	2,000	346	73	29
C-reactive protein, mg/dL	6.54	2.82	0.89	0.44
Lactate dehydrogenase, U/L	1,913	362	209	178

## Discussion

HLH is an extremely aggressive and life-threatening condition associated with excessive activation of the immune system and subsequent multi-organ dysfunction. It was first mentioned by Farquhar and Claireaux in 1952 [6]. There are two types of HLH, primary and secondary. Primary, also known as the familial type, is commonly seen in children and is caused by genetic defects involving cytotoxic pathway of perforins. By contrast, the secondary form, also known as the acquired type, can occur in any age group. The secondary type is seen in patients with predisposing viral infections. The most common triggering viruses are EBV; parvovirus B19; human immunodeficiency virus (HIV); hepatitis A, B, and C; and adenovirus [3,7]. It is also seen in patients with underlying malignancies or rheumatological or autoimmune diseases. Since the spread of SARS-CoV-2, COVID-19 has been identified as one of the triggers of secondary HLH [8]. During SARS-CoV-2 infection, the pathogenesis is believed to be subclinical inflammation causing macrophage activation and modulation.

The most common presenting findings in HLH are fever, fatigue, hepatosplenomegaly, neurological symptoms, and rash. HLH diagnosis is based on the HLH 2004 study diagnostic criteria: It should include either molecular diagnosis with pathologic mutations of PRF1, STX11, UNC13D, SH2d1A, Munc18-2, Rab27a, or BIRC4, or five out of the following eight criteria should be fulfilled: fever  > 38.5°C; splenomegaly; peripheral blood cytopenia (bicytopenia/pancytopenia); hypertriglyceridemia >265 mg/dL and/or hypofibrinogenemia  > 150 mg/dL; low or absent natural killer cell activity; ferritin > 500 ng/mL; hemophagocytosis in bone marrow, spleen, lymph node, or liver; and elevated soluble CD25 >2SD (>2 standard deviations above the mean) [4,9,10].

As HLH has a high mortality without appropriate treatment, all these criteria are not always required for diagnosis, as some of the diagnostic criteria can develop late in the disease course. Hence, if there is a high clinical suspicion, it is important to consider diagnosis and start management. There are also modified criteria that can be sufficient to diagnose HLH, which include three of the following four findings: fever, cytopenia, splenomegaly, and hepatitis-along with abnormality in one of the following four markers: increased ferritin, hypofibrinogenemia, hemophagocytosis, and absent or decreased natural killer cells [10]. Our patient had fever, cytopenia, hepatosplenomegaly, increased ferritin levels, and a high CD25 level. He fulfilled five out of eight criteria, suggesting the most likely diagnosis is HLH.

The H-score helps in calculating probability of having HLH. First mentioned by Fardet et al., it includes fever, hepatosplenomegaly, cytopenia, hyperferritinemia, transaminitis, hypofibrinogenemia, hypertriglyceridemia, underlying immunosuppression, and hemophagocytes on bone marrow aspirate. The H-score of 169 is considered as a cut-off for considering HLH [5]. Our patient had a score of 174, indicating a 54%-70% probability of HLH.

HLH during the acute phase of COVID-19 infection has been mentioned in various case reports [11,12]. However, post-COVID-19 HLH cases are rare. Subclinical inflammation post-COVID-19 infection leading to immune dysregulation and activation of macrophages is thought to underlie the pathogenesis of HLH in post-COVID-19 patients [13-15]. Without appropriate management, patients with HLH only survive for a few months. HLH treatment is based on the HLH 94 protocol, which includes eight-week induction therapy with etoposide and dexamethasone, with or without intrathecal methotrexate [16]. The principal goal of induction therapy is to suppress the life-threatening inflammatory process that underlies HLH. After eight-week induction therapy, based on the patient’s clinical response, therapy is weaned off/discontinued or transitioned to continuation therapy, which can act as a bridge to bone marrow transplantation (BMT). BMT is recommended for patients with refractory disease or relapse, persistent natural killer cell dysregulation, or proven underlying genetic/familial disease [17].

Our patient showed significant response to induction therapy with etoposide and dexamethasone, with improvements in hemoglobin, platelet count, ferritin, and transaminase levels (Table 1).

## Conclusions

A timely diagnosis of HLH plays a vital role in the appropriate management of patients with HLH. It is also crucial to identify underlying triggers such as viral infection. Since the onset of the COVID-19 pandemic, many post-COVID-19 complications have been reported, including myocarditis, pulmonary fibrosis, and thrombotic thrombocytopenic purpura. HLH has been identified in an increasing number of patients in the acute phase of COVID-19 infection, but reports of post-COVID-19 HLH are rare. The present case study of post-COVID-19 secondary HLH, which was diagnosed in a timely manner, has demonstrated significant improvement of the disorder with induction therapy.
